# CO-IRv2: Optimized InceptionResNetV2 for COVID-19 detection from chest CT images

**DOI:** 10.1371/journal.pone.0259179

**Published:** 2021-10-28

**Authors:** M. Rubaiyat Hossain Mondal, Subrato Bharati, Prajoy Podder

**Affiliations:** Institute of Information and Communication Technology, Bangladesh University of Engineering and Technology, Dhaka, Bangladesh; University of Engineering & Technology, Taxila, PAKISTAN

## Abstract

This paper focuses on the application of deep learning (DL) in the diagnosis of coronavirus disease (COVID-19). The novelty of this work is in the introduction of *optimized InceptionResNetV2* for COVID-19 (CO-IRv2) method. A part of the CO-IRv2 scheme is derived from the concepts of InceptionNet and ResNet with hyperparameter tuning, while the remaining part is a new architecture consisting of a global average pooling layer, batch normalization, dense layers, and dropout layers. The proposed CO-IRv2 is applied to a new dataset of 2481 computed tomography (CT) images formed by collecting two independent datasets. Data resizing and normalization are performed, and the evaluation is run up to 25 epochs. Various performance metrics, including precision, recall, accuracy, F1-score, area under the receiver operating characteristics (AUC) curve are used as performance metrics. The effectiveness of three optimizers known as Adam, Nadam and RMSProp are evaluated in classifying suspected COVID-19 patients and normal people. Results show that for CO-IRv2 and for CT images, the obtained accuracies of Adam, Nadam and RMSProp optimizers are 94.97%, 96.18% and 96.18%, respectively. Furthermore, it is shown here that for the case of CT images, CO-IRv2 with Nadam optimizer has better performance than existing DL algorithms in the diagnosis of COVID-19 patients. Finally, CO-IRv2 is applied to an X-ray dataset of 1662 images resulting in a classification accuracy of 99.40%.

## 1. Introduction

In late 2019, China first reported *novel coronavirus disease* (COVID-19) caused by *severe acute respiratory syndrome coronavirus 2* (SARS-CoV-2) [1]. In March 2020, the World Health Organization (WHO) declared COVID-19 as a pandemic. Some common symptoms related to COVID-19 include cough, fever, dyspnea, and sore throat [2–4]. The respiratory droplets and the interaction between human-to-human are the leading causes of the spread of this infection [5]. The incubation time of COVID-19 is significantly longer, causing difficulty in the control of this pandemic. Moreover, some COVID-19 patients are asymptomatic, which causes the spreading of the infection [6]. Due to these causes, the early recognition, diagnosis, and control of infectious diseases like COVID-19 are considered hard.

Efforts have been made to find an efficient method for diagnosing COVID-19. RT-PCR test is an authorized method for detecting COVID-19 using a nasopharyngeal or oral swab [7]. The RT-PCR test is time-consuming and complex. Besides, RT-PCR test has some limitations, which result in insufficient sensitivity and a higher false-negative rate [8, 9]. Several patients who are incorrectly detected as negative are likely to interact with a massive number of people. Hence, a superior diagnosis technique with a low false-negative rate is required to control the risk of COVID-19. Nevertheless, radiology imaging, i.e., X-rays [10], ultrasound, magnetic resonance imaging (MRI), and computed tomography (CT) image scanning are also applied to diagnose various diseases, including COVID-19 [10–18]. Sometimes underlying pathology may hide viral infection, such as in patients with pulmonary fibrosis. The diagnosis of COVID-19 is considerably hard. However, sometimes X-rays can provide a false-negative rate. In contrast, chest CT scan images provide detail view useful for the rapid diagnosis of COVD-19. This also helps in the timely isolation of infected patients and thus managing COVID-19 [2, 19–21].

Concisely, the early and accurate diagnosis and treatment of COVID-19 suspects can play a significant role in treating patients. This is important for the COVID-19 control, prognosis of patients, and the security of public health. Chest CT can detect small areas of ground-glass opacity (GGO) for its high resolution. In the early phases of COVID-19-pneumonia, pulmonary outcomes in lung CT images may be present with peripheral, subpleural, and minor GGOs [22], which may take much longer than large-involved and isolated GGOs or/and patterns of integration. Moreover, radiologists’ visual fatigue will increase the possible risks of missing a diagnosis for several minor lesions. Hence, it is essential to develop an artificial intelligence (AI) system for the computer-aided diagnosis of COVID-19.

As a subset of AI, deep learning (DL) has been applied to automatically detect respiratory diseases and illnesses [23–28]. However, lesions need to be distinguished for most DL-based approaches for the diagnosis of CT segments. Harmful lesions of COVID-19 cost much effort for radiologists, while COVID-19 spreads rapidly and is not adaptable for radiologists. One of the most accessible labels to diagnose COVID-19 is the patient-level label, where patients are labeled negative or positive. An appropriate DL-based scheme can be crucial for the diagnosis of COVID-19. Hence, this work explores a DL model for the automatic diagnosis of COVID-19.

The main contributions of this paper are summarized as follows:

- A new approach termed as Optimized InceptionResNetV2 for COVID-19 (CO-IRv2) is proposed in this work. Hyperparameter tuning is conducted for the optimization of CO-IRv2.
- A dataset of 2481 CT images is formed by combining two different databases.
- The proposed CO-IRv2 is applied to the new dataset of 2481 CT images and an existing dataset of X-ray images. The effectiveness of several optimizers in CO-IRv2 is evaluated in classifying normal people and suspected COVID-19 patients.

This paper is structured as follows. The background of these studies is introduced in Section 2. Moreover, in Section 3, dataset descriptions are briefly presented. Furthermore, methodology and experimental discussions are provided in Section 4 and Section 5, respectively. Finally, the concluding remarks are provided in Section 6.

## 2. Literature review

A number of research work proposed various DL models for detecting or diagnosis of COVID-19. These studies are summarized in Table 1 and are described in the following.

**Table 1:** Summary of literature reviews for CT images using DL.

Ref.	Methods	No. of images	Number of classes	Performances	Limitations
[30]	ResNet18	618	3	Recall:81.50%, Accuracy:86.70%, F1-Score:81.10%, Precision = 80.80%	The limited number of samples
[29]	ResNet50	495	2	Recall:81.10%, Accuracy:76%, AUC:81.90%, Specificity:61.50%	Using just slices of the lung area may lead to misdiagnosis
[31]	Modified ResNet 50 V2	63,849	2	Accuracy: 98.49%, recall: 96.83%	Considered only CT images
[32]	VGG-19	812	2	Accuracy:94.50%	Only used RMSProp optimizer for few CT images
[33]	U-Net CNN	540	2	Specificity: 91.10%, Recall: 90.70%, and AUC:95.90%	Lung segmentation had no temporal information, data from only a single hospital
[34]	2D CNN	1065	2	Recall: 87%, specificity:88% and accuracy: 89.50%	Training dataset was marginal
[35]	U-Net++	44	2	-	Selection bias and small sample size
[36]	2D and 3D Deep learning models, U-Net Model	157	2	Recall:98.20%, AUC: 99.60%, and Specificity:92.20%	The study used a limited number of CT scan images
[37]	2D U-Net	1,230	2	F1 score: 97.32%	Only considered CT images, and a small dataset
[38]	DRENet	1,485	2	Accuracy: 94%, Precision: 96%, recall: 93%, F1-Score: 94%	The study used a limited number of CT scan images
[39]	EDL_COVID	7500	3	Accuracy: 99.05%, F1-score: 98.59%, recall: 99.05%,	Considered only CT images
[40]	InceptionV3	746	2	Accuracy: 82%, precision: 82.50%, recall: 81.40%, F1-score: 81.50%	Only considered CT images, and a small dataset
[42]	CRNet	746	2	AUC:94%, F1-Score:85%, Accuracy:86%	Low number of training CT images
[43]	Multi-layer perceptron with Encoder Decoder	1044	2	Specificity:79%, AUC:93%, Recall:94%, Accuracy:86%	Only considered CT images, and dataset of patients is small in number
[46]	Multi-task learning and single-task learning	408	2	AUC:86%±2%, Specificity:88%±1.50%, Recall:77%±3%, Accuracy:86%±2%	Only considered CT images, and a small dataset
[47]	Elastic Net	1381	2	AUC:94%, Recall:79%, Accuracy:90%, Specificity:91%	Considered only CT images

Wu et al. [29] proposed a framework of coronavirus screening based on ResNet50, a modified form of CNN. The dataset was collected from two hospitals where the images of COVID-19 and other cases of pneumonia were 368 and 127 images, respectively. All images were resized to a size of 256×256 by using image augmentation. They achieved a specificity of 61.5%, a recall of 81.1%, an accuracy of 76% and an area under the receiver operating characteristics curve (AUC) of 81.9% after preprocessing. The authors of [29] created a validation, training, and testing set using just slices of the maximal lung area in three perspectives. Additional CT slices in various perspectives were also provided as input to the suggested model to increase its performance. Other clinical data, such as body mass index and severity of COVID-19 infection, were not available for subgroup analysis. Nonetheless, this study’s age and gender subgroup analyses enabled an initial assessment of the model. Despite the importance of this work, more powerful AI algorithms need to be created to facilitate and accurately diagnose COVID-19. The model in [29] could not minimize misdiagnosis and thus affected clinical outcomes.

The researchers of [30] classified different types of diseases like Influenza-A viral pneumonia and COVID-19 pneumonia from CT images using CNN variants such as ResNet18. They used 618 CT images from three different datasets provided by different hospitals. Their proposed model provided 80.8% precision, 81.5% recall, 86.7% accuracy and 81.1% F1-score [30]. As reported in [30], the symptoms of COVID-19 could overlap with those of other pneumonia, including eosinophilic pneumonia, organizing pneumonia and influenza-A viral pneumonia (IAVP). The study in [30] compared the CT manifestations of IAVP and COVID-19. One limitation of the work was the use of limited number of samples. Moreover, the work [30] did not address the complicated clinical condition and further multicenter clinical trials.

The dataset of the work of modified ResNet50v2 [31] included 48,260 CT scanning images of 282 normal people and 15,589 images of 95 COVID-19 patients. The initial stage in the image processing technique was to evaluate the view of the lung and discard any CT images that were not properly visible in the lung. This significantly lowered processing time and false detections. The authors then created a novel architecture to categorize the ResNet50v2 model over a range of image resolutions, ensuring that the model did not lose data from small objects. The system evaluated the patient’s condition using a predefined threshold. The authors tested their system in different methods using ResNet50v2, Xception, and modified ResNet50v2 [31].

In [32], a self-built model named CTnet-10 was developed with an accuracy of 82.1% for the diagnosis of COVID-19. Additionally, the authors experimented with other models, including VGG-16, VGG-19, ResNet-50, DenseNet169, and InceptionV3. With an accuracy of 94.52%, the VGG-19 outperformed all other DL models considered in the study. The work [32] only used RMSProp optimizer for few CT images. U-Net CNN architecture was proposed to detect COVID-19 from 540 images [33], where the U-Net was applied for lung segmentation. The results of the segmentation were provided as the 3D-CNN input for the prediction and possibility of COVID-19. Their models provided 91.1% specificity, 90.7% recall, and 95.9% AUC [33]. One limitation was that the lung segmentation did not include temporal information and was trained using imprecise ground-truth masks. Another limitation was that the research data were derived from a single hospital, and no cross-center validations were conducted.

Moreover, the work [34] proposed a 2D CNN model for scanning viral pneumonia and COVID-19 from 1065 CT images. The authors of [34] also modified the inception DL algorithm to establish the model, followed by external and internal validation, where external validation showed 83% specificity with 79.3% accuracy. On the other hand, internal validation obtained 87% recall, 88% specificity, and 89.5% accuracy [34]. Several factors, including low signal to noise and complex data integration affected the efficacy of the DL used in [34]. Because of the relatively large amount of CT scan parts, especially those irrelevant for diagnosing pneumonia, classification was a difficult task.

The authors of [35] considered CT images and built a U-Net++ segmentation model to detect COVID-19. They invented the VB-Net method for extracting lung regions and infected lungs. They provided accurate medical research quantification data, including a quantitative assessment of progression and disease prediction. The study obtained successful performance by including the human loop method into the development of a segmentation network based on the VB-Net. The drawbacks of this study were its retrospective nature, selection bias (absence of severe COVID-19 patients), small sample size, and evaluation bias in the radiologist-defined CT score. Another study considered 2D and 3D U-Net models for COVID-19 detection [36]. In addition, the authors of [37] suggested a 2D DL architecture with a U-Net backbone to detect lung areas. The authors conducted two-division tasks: the first was a segmented abnormality in the chest CT scan specific to COVID-19 infection, and the second was a segmented CT lung space. Together with the volumetric assessment, the two segmentation tasks enabled a chest CT scan prediction to give statistical information on COVID-19 anomalies. The authors got 84.7% of mean intersection over union (M-IoU) and an F1 score of 97.32%. For semantic segmentation, their research utilized lung CT image sets from Kaggle and GitHub.

The work of [38] automatically extracted radiographic features indicating developing pneumonia from a radiograph, most notably the GGO. They developed a model of Deep Pneumonia to assist doctors in recognizing and diagnosing COVID-19 pneumonia. Their strategy was divided into three phases. First, they isolated the damaged lung areas and used lung segmentation filter banks to eliminate any noise. The second step utilized the deep temporal regression network (DTRN) to extract top characteristics from CT images and to provide picture-level predictions. Image-level predictions were aggregated in the last stage to generate a patient-level diagnosis. Their model was based on 88 COVID-19 patients and 100 patients with bacterial pneumonia from 777 image datasets. They achieved an AUC of 0.95 for DRE-Net, which is much higher than the AUCs reported for other models such as ResNet, VGG16, and DenseNet. The study’s drawback was the use of a small number of CT scan images to detect COVID-19.

The authors of [39] developed a DL ensemble model for identifying COVID-19 CT images. A total of 2933 COVID-19 lung CT pictures were gathered from public sources, past publications, and major media stories. The images were preprocessed in order to get 2500 images of exceptional quality. A hospital obtained 2500 CT scans of a lung tumor and 2500 normal lung images. Transfer learning was used to establish the model parameters, and three deep convolutional network models were pre-trained: GoogleNet, ResNet, and AlexNet. All the images were extracted using these models. Softmax was used to classify the layers in a completely linked manner. A relative majority vote was used for classification of the images.

In one study, a total of 15 pre-trained CNN architectures were used: ResNet-50, ResNext50, SeResnet 50, DenseNet121, EfficientNets(B0-B5), Xception, NasNetMobile, NasNetLarge, InceptionV3, and Inception ResNetv2 [40]. Utilizing the optimum mix of deep transfer learning output, the work then created a group method based on a majority vote to further improve the classification performance. The authors of [40] analyzed a publicly available dataset of CT scans that included 349 CT scans categorized as COVID-19 positive, and 397 CT scans having COVID-19 negative samples [40].

Furthermore, the authors of [41] established a DL method named CovidCTNet to diagnose COVID-19 infection from CT images. This work applied U-Net model for developing BCDU-Net architecture. This algorithm could distinguish CAP, COVID-19, and control lungs from CT images. They used 89145 images with 32230 CT images of COVID-19 confirmed patients, 25699 images of CAP, and 32216 images of other disorders or healthy lungs. For the evaluation, the holdout method was used with 90% of data samples used for training, and 10% for testing. Their proposed method provided a recall of 87.5%, an AUC of 95%, a specificity of 94%, and an accuracy of 91.66% [41]. A novel DL method CRNet was proposed for COVID-19 detection from 746 CT images, where the images from three open-access datasets were divided into 60% training, 25% testing, and 15% validation [42]. The method achieved a value of 94% AUC, 85% F1-score, and 86% accuracy [42].

In [43], a DL scheme containing a multi-layer perceptron, two decoders, and an encoder was applied to 1044 images [43]. The number of COVID-19, healthy individuals, lung cancer patients and different types of pathology were 449, 100, 98 and 397, respectively. The data was split into 80% training, 10% testing and 10% validation. This system obtained the highest AUC, recall, specificity and accuracy of 93%, 94%, 79% and 86%, respectively [43]. Aside from the numerous advantages of using CT images to identify and isolate early COVID-19 patients, DL methods based on CT images can also aid physicians in combating this disease [44]. This is because DL can be used not only to segment and classify images in the field of healthcare, but also to predict treatment outcomes [44]. Another effective technique is to utilize unsupervised learning algorithms to identify the picture and identify lesions when only a limited dataset is accessible [45]. These marginally better tactics could help fight against the coronavirus COVID-19, where just a few databases are usually available, and clinicians cannot provide a big volume of labeled data.

Zhu et al. [46] proposed DL methods where they used multi-task learning and single-task learning. They used 408 real CT images from two different sources. They achieved 85.91% accuracy for their proposed best model [46]. However, the focus of that study was to predict whether the patient would develop severe symptoms based on mild symptoms. That would result in a classification imbalance because only a small number of patients would progress to severe conditions. First, the imbalanced categorization of the dataset was biased, with 86 severe instances compared to 322 non-severe instances. That complicated the process of developing good classification models [46]. Moreover, another DL model, Elastic Net [47] segmented 1224 images for training and testing, where the total images were 1381, including 1200 non-COVID-19 images and 181 COVID-19 images. The combination of 3D and 2D CNN was used for segmentation purposes. Next, the dataset was evaluated by Elastic Net. This model obtained an AUC of 88.2%, while the testing data had 641 patients. The highest AUC, recall, accuracy and specificity were 94%, 79%, 90% and 91%, respectively [47].

Narin et al. obtained 96.10% accuracy when they applied ResNet50 model to 3141 X-ray images where 341 are of COVID-19 patients, and the remaining are of normal people [48]. One study [49] considered a dataset of 224 COVID-19 X-ray images and obtained a classification accuracy of 98.75% using pre-trained DL methods. Another study used ResNet50 along with a support vector machine to 381 X-ray images resulting in an accuracy of 98.66% [50]. A DL model achieved 98.08% accuracy when applied to detect COVID-19 patients from an X-ray dataset where 127 cases are for COVID-19 patients [16].

The above studies reveal that the lack of large COVID-19 datasets is challenging to validate different DL models. Some of the existing models described above were only tested for CT images, so their effectiveness in the case of X-ray images was not reported. Some others only focused on X-ray images without considering CT images at all. Furthermore, most of these works split image samples using the holdout method without considering the cross-validation method. Hence, this paper generates a new dataset of 2481 CT images by combining two different databases. This paper proposes a new DL algorithm CO-IRv2, and its performance is evaluated for CT images and X-ray images. Furthermore, this paper considers the holdout method as well as cross-validation methods for splitting the training and testing images.

## 3. Description of database

**Fig 1:**
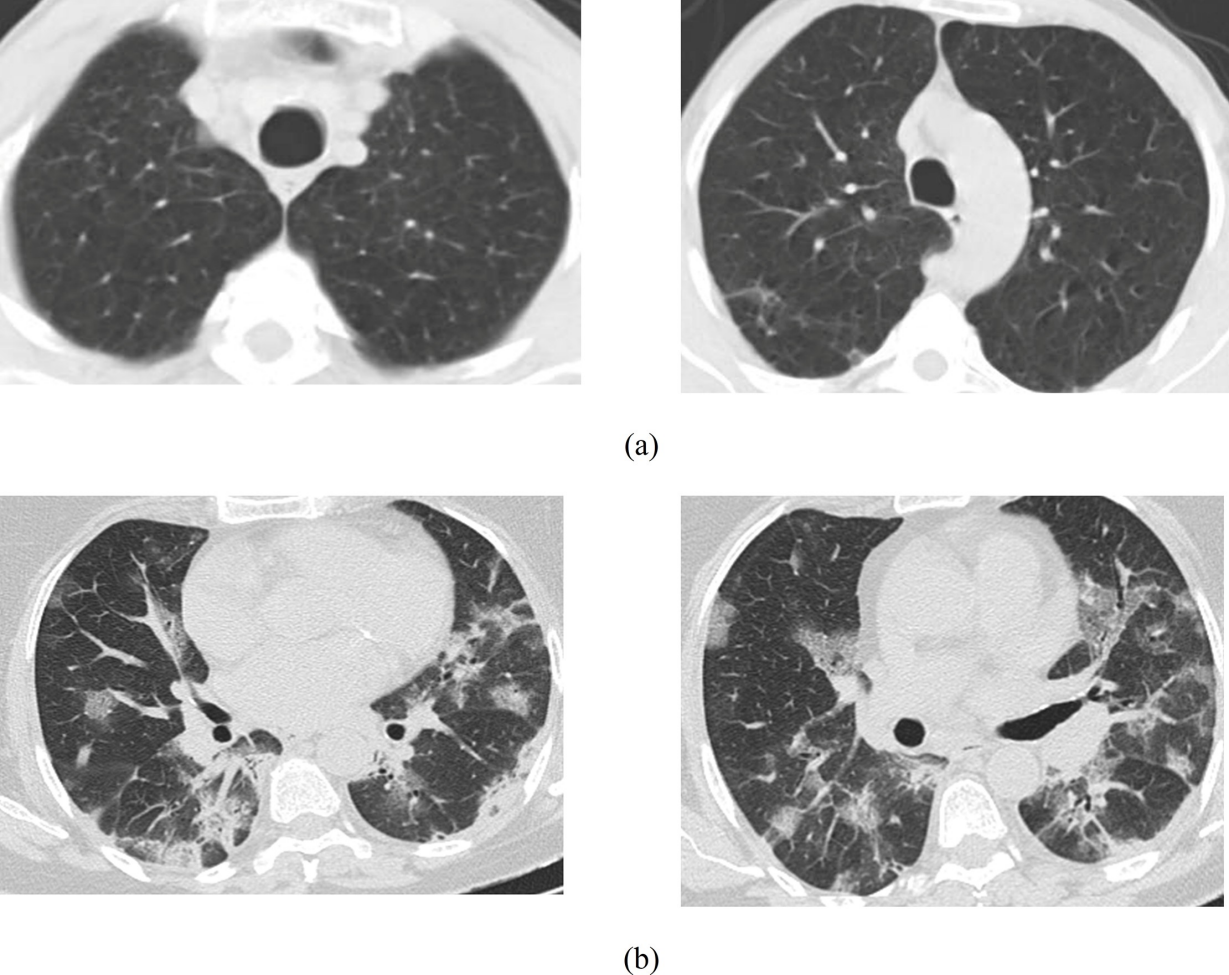
Sample of the (a) non-COVID-19 and (b) COVID-19 CT scan dataset [53].

Our experimental dataset was retrieved from two different open access sources [51, 52]. The dataset of [51] had 829 CT images. On the other hand, the dataset of [52] had 2482 CT images where normal and COVID-19 patients were 1230 and 1252, respectively. After collecting the CT images, we formed a database where we retrieved the 829 images from the dataset reported in [51], and the rest of the images were retrieved from the dataset available in [52]. The total CT images for our experiments were 2481, where the images of normal and COVID-19 patients were 1229 and 1252, respectively. The resultant dataset is now made available in [53]. Fig 1 shows samples of our generated dataset [53], Fig 1(A) is for non-COVID and Fig 1(B) is for COVID-19 cases. Next, we split our database into training and testing. The details of the training and testing samples are shown in Table 2. This work also considers a dataset of X-ray images [54] containing 1662 images, of which 1583 images are of normal people and 79 are of COVID-19 patients.

**Table 2:** The number of CT images per class applied in training and testing stages.

Split	Normal	COVID-19
Training	971	1013
Testing	258	239
Total	1229	1252

## 4. Methodology

**Fig 2:**
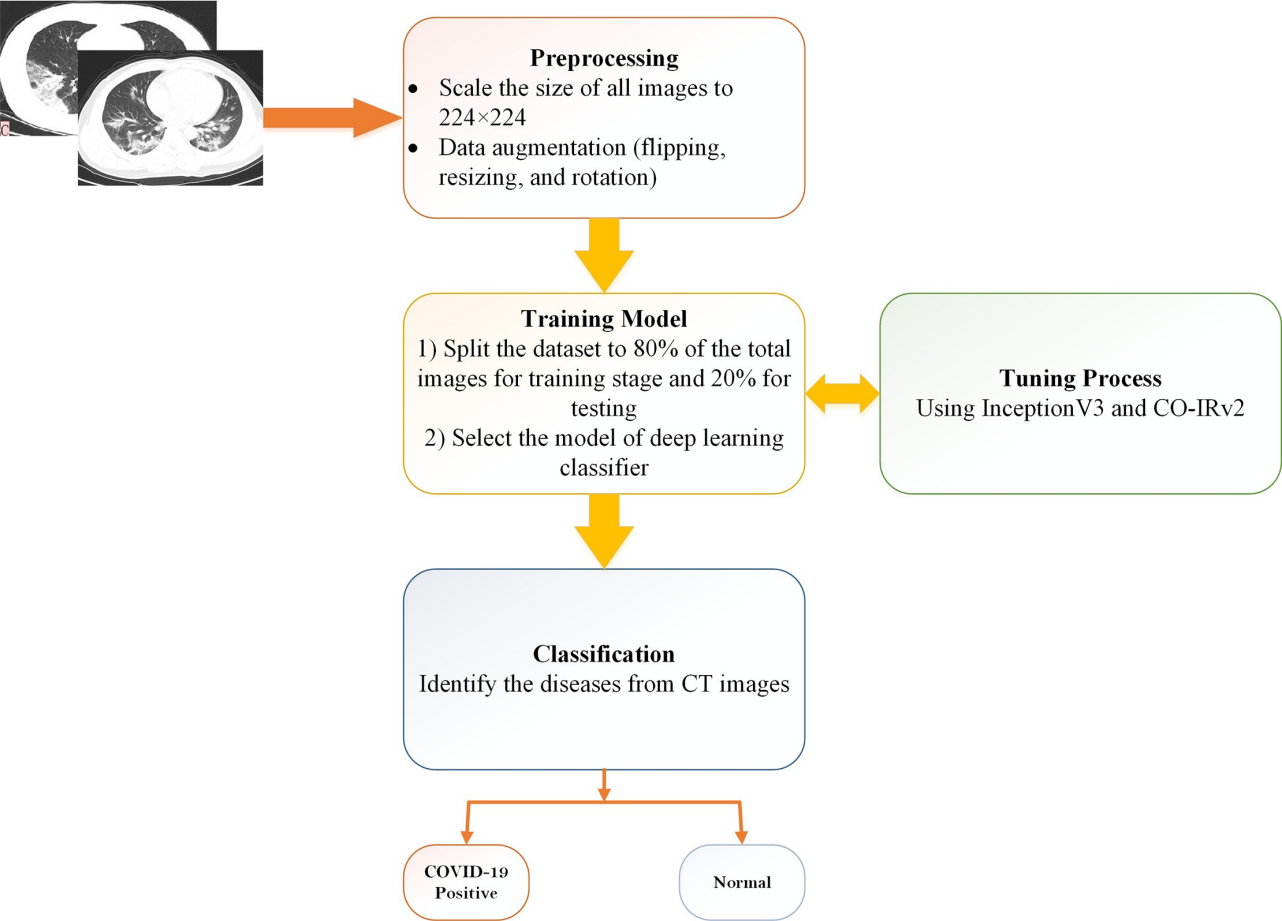
Basic summary of our proposed work.

The experimentation had several stages. Data resizing and normalization were performed to facilitate generalization and avoid overfitting. The dataset was divided into two parts: training and testing. The proposed CO-IRv2 model was trained using training data. The evaluation was run up to 25 epochs. The proposed model got the desired accuracy within 25 epochs. Next, we performed hyperparameters’ fine-tuning on the proposed model. The CO-IRv2 model avoided underfitting by using several dense layers described in Section 4.2. Moreover, CO-IRv2 avoided overfitting of the model by using regularization techniques: data augmentation described in Section 4.1, and dropout discussed in Section 4.2. Afterwards, the system was evaluated with respect to precision, recall, F1-score, confusion matrix, receiver operating characteristics curve (ROC) curves, and accuracy. A basic system of our proposed work is depicted in Fig 2.

### 4.1 Data Preprocessing

Several steps, including normalization and data augmentation, were taken to tune the hyperparameters of CO-IRv2. Normalization of the data is a critical step to guarantee numerical stability. Normalization accelerates the training of a model and increases the likelihood of steady gradient descent. The samples of the CT images were of different sizes. Hence, we resized the images to 224×224 pixels with RGB color. The pixel values of the input images were normalized to a value between 0 and 1. The data sets include grayscale photos that have been rescaled by multiplying 1/255 by the pixel values. Next, we applied data augmentation [51, 52] that increases the sample size as models need a wide range of data for successful training. However, in many cases, the number of CT images in a dataset is less, since collecting medical data is difficult. Data augmentation increases the number and the variability of images while maintaining class labels. In this work, the CT images in the dataset were extended by applying the following methods: (1) the images were rotated by angles of 180 degrees clockwise, (2) the images were scaled by 15%, (3) the images were horizontally rotated and (4) Gaussian noise with a mean zero and variance 0.25 were added. Besides applying flipping, resizing, and rotation, we applied random horizontal flips to improve the simplification of the architecture for each probable location of CT images. Finally, a number of images were produced by using a 180-degree rotation. In data augmentation, random horizontal flip helped in the identification of COVID-19 from chest symptoms and random resized crop helped in deeper recognition of pixels’ relationship by varying image intensity. The mentioned augmentation and development strategies were applied to promote the generalizations of the proposed model. It should be noted that all these methods were used for training samples. Finally, there was a bigger training set of 2481 images, 5 times more than the initial training images.

### 4.2 Proposed CO-IRv2

This section discusses the proposed CO-IRv2 algorithm. A part of the CO-IRv2 scheme is derived from the concepts of Inception and ResNet [55] with hyperparameter tuning, while the remaining part is a new architecture consisting of a global average pooling layer, batch normalization, dense and dropout layers.

**Fig 3:**
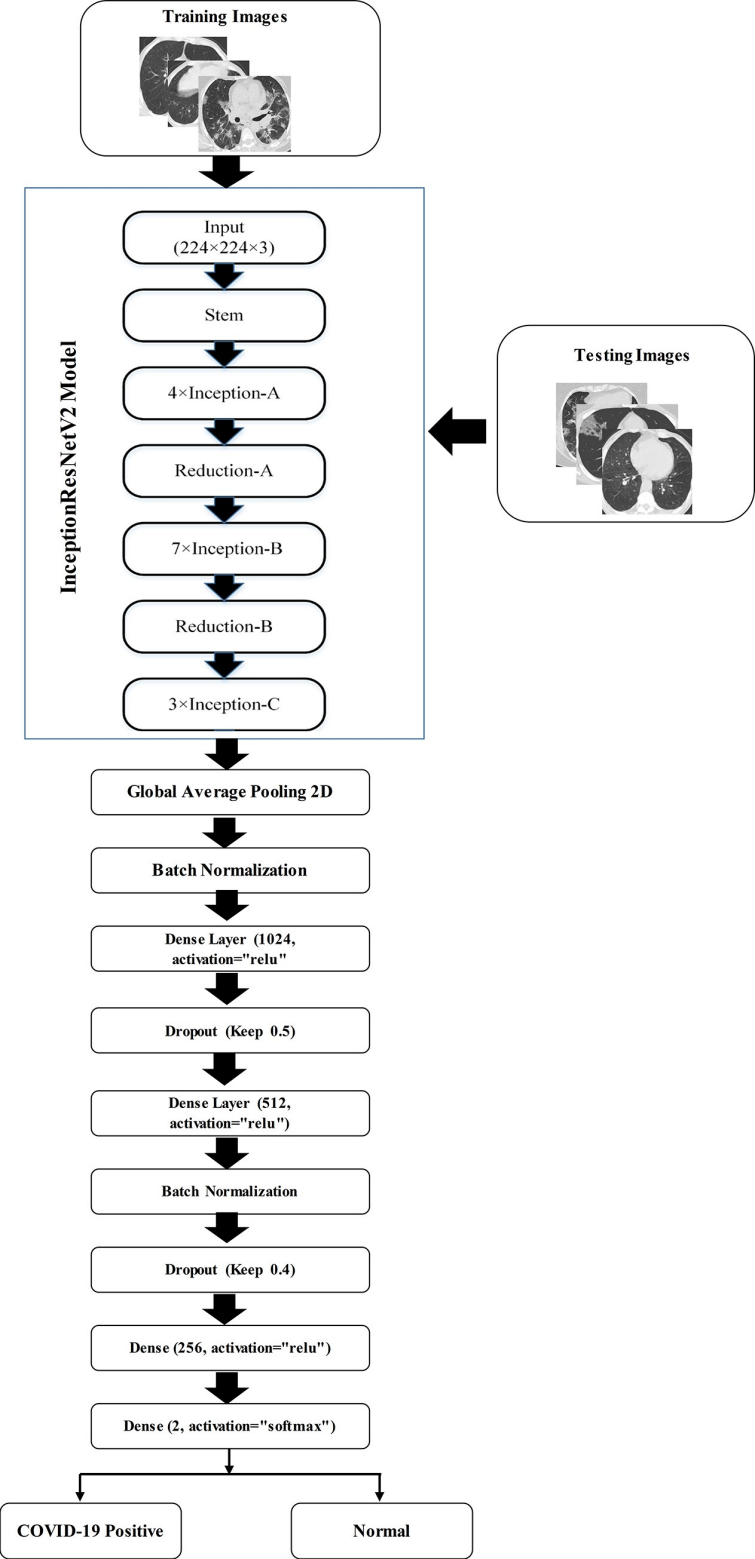
Sample diagram of the proposed CO-IRv2.

Fig 3 shows the CO-IRv2 architecture where the upper part (within the rectangular box) is derived from InceptionNet and ResNet, while the lower part is a new arrangement of multiple layers. In the upper part, there are the stem, Inception [56], and reduction modules. The Inception model is substantially tunable, which means some changes like changing the number of filters and layers have to be performed without impacting the quality of the fully connected layers. By carefully tuning the sizes of the layers, the training speed can be optimized. For our proposed CO-IRv2 model, uniform selections are performed for the Inception modules for all grid sizes. In CO-IRv2, batch-normalizations are applied distinctly on top of the conventional layers, instead of above the combinations of InceptionNet and ResNet. CO-IRv2 is based on Inception architecture having residual connections instead of filter concatenation operation. The Inception module selects all three filter sizes at each layer instead of choosing one for each layer. The use of multiple filters enables the selection of the best features resulting in an excellent performance. The addition of ResNet and Inception allows the model to have excellent accuracy than standalone Inception and ResNet models. In CO-IRv2, the residual connectors are used to combine different convolution filters. The residual connectors cause a reduction in the computation time during the training phase. Specifically, the residual blocks are utilized to permit that the Inception modules can increase their quantity. Hence, the depth of the network can also be increased. The extremely deep networks of CNN is the training stage, where the computation time is reduced by the residual networks [55, 56]. When more than 1,000 filters are deployed in the network, the network reduces the residue to solve the training problem [56].

The stem, Inception and reduction modules consist of convolution layers, pooling layers and Rectified-Linear-Unit (ReLU) activation functions. Convolution layers are applied to generate features from images. A convolution kernel operates on the input images by sliding across them. This is done with a set of movement parameters known as a stride. The size of the output of convolution operation depends on the size of the kernel and that of the slide. Optimization is done on the bias and the weights of the kernels. The activation layers enhance the nonlinearity of the output obtained from the convolution layers. The activation function is a decision-making function that aids in the learning of complex patterns. The use of the right activation function can speed up the learning process. ReLU is an activation function having a fast calculation speed. Pooling layers are used to reduce the feature map. Pooling is a form of local operation that processes similar data in the vicinity of the receptive field. Pooling layers also reduce the upper layers in terms of the number of parameters and size in space. These pooling layers help the CO-IRv2 model to remain invariant to the effects of translation, distortion and transformation. The stem, Inception and reduction elements are further described with illustrations later in this section.

Next, we describe the lower part of the CO-IRv2 architecture, as shown in Fig 3. Firstly, there is a global average pooling layer where a pooling operation *g*_*p*_(.) generates feature map $Q_{l}^{k}$ from input feature-map $P_{k}^{l}$ as follows.


$$Q_{l}^{k}=g_{p}(P_{k}^{l})$$ (1)

The next operation is the batch normalization that addresses the issues of internal covariance shift inside feature maps. It smoothes the gradient flow and works as a regulatory component, so assisting in the network’s generalization. The normalized feature map $R_{l}^{k}$ for a mini-batch can be represented as follows.

$$R_{l}^{k}=\frac{P_{l}^{k}-\mu }{\sqrt{\sigma_{B}^{2}+\varepsilon }}$$ (2)

where *μ* is the mean and $\sigma_{B}^{2}$ represents the variance of a feature-map, and the term *ε* is considered to address the divide by zero issue. After that there are multiple dense layers that are associated with a leaky ReLU and a dropout layer. These dense layers contribute to improving classification accuracy. A minor negative side is allowed by a leaky ReLU. To keep a regulated negative component, leaky ReLU activation with a value of 0.2 is used for each dense layer. The dropout process sets the output of each hidden neuron to zero with a probability of half. The neurons that are dropped out in this fashion do not participate in forward or backpropagation. Consequently, the neural network samples a different design each time an input is supplied, yet all of these structures have the same weights. Dropout layers are used for regularization by randomly dropping out or ignoring some layer outputs or units. Dropout layers can reduce the effect of over-fitting. This is done by reducing the number of parameters of the model. The dropout layer increases the robustness of the CO-IRv2 framework. Fig 3 shows one dropout layer in CO-IRv2 configured at rate 0.5, while the other with a rate of 0.4. Softmax layers are used to transform the input values into probabilities so that the sum of the values becomes unity. The softmax function is most typically employed as an activation function in a neural network model. Softmax is used as the activation function for multi-class classification problems that need class membership on more than two class labels. In this way, softmax enables multi-class classification. Softmax is a mathematical function that converts an integer vector into a probability vector. This is done by ensuring the probability of each value is proportional to the relative scale of the vector.

**Fig 4:**
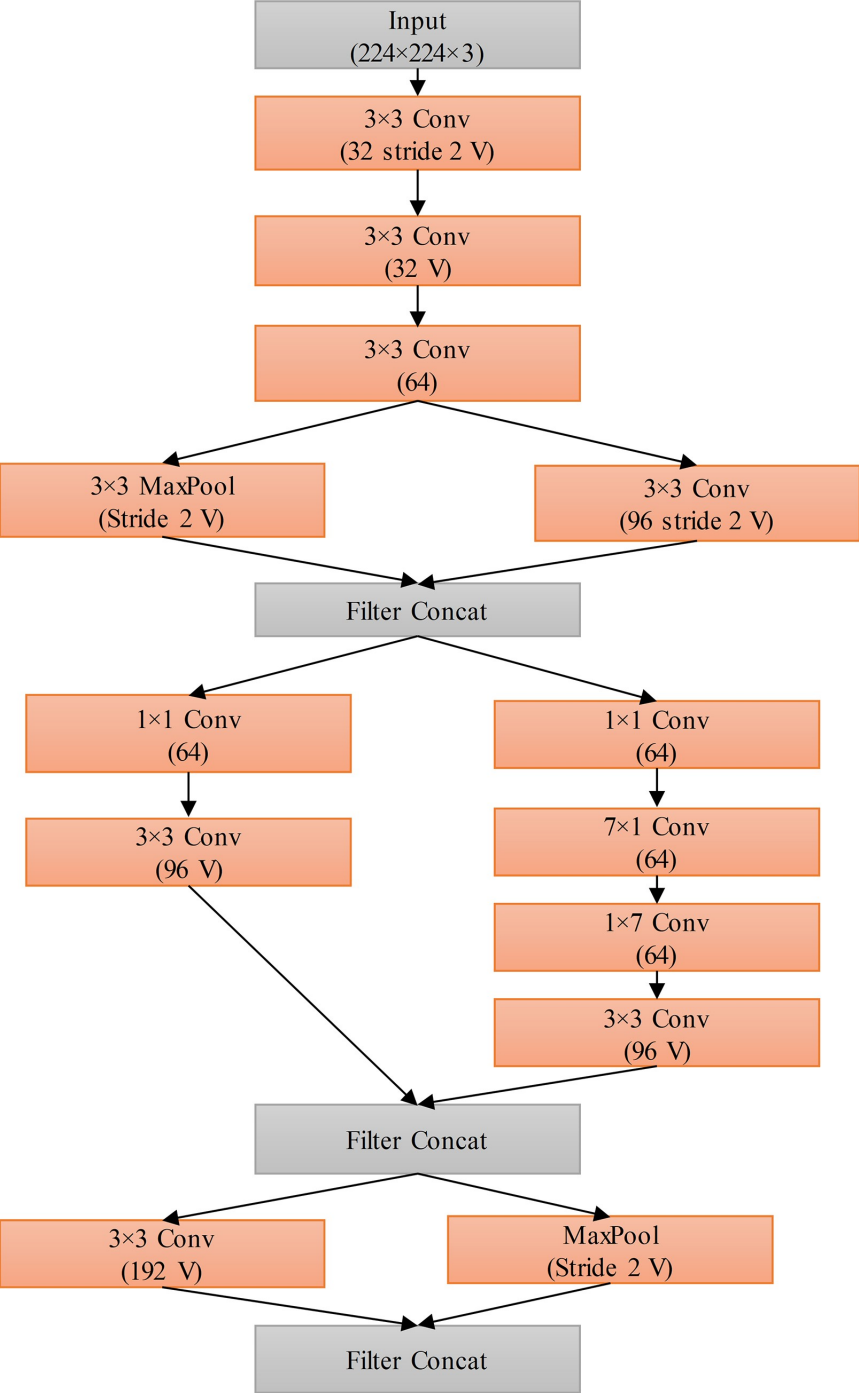
Stem diagram of CO-IRv2.

Fig 4 describes the details of the stem in CO-IRv2. It can be seen that the initial blocks within the stem compute three 3×3 convolutional operations on the given input. Next, there are three inception blocks. The first one has two paths, one for 3×3 convolutional operations and the other for max pooling. The two paths are concatenated and passed to the next inception block. The second inception block also has two paths; one path has 1×1 and 3×3 convolutional operations, while the other has 1×1, 7×1, 1×7 and 3×3 convolutional operations. The output of the two paths is concatenated. Similar to the first block, the third inception block has two paths, one for 3×3 convolutional operations and the other for max pooling. The final output is the concatenation operation of the two paths within the third inception block.

**Fig 5:**
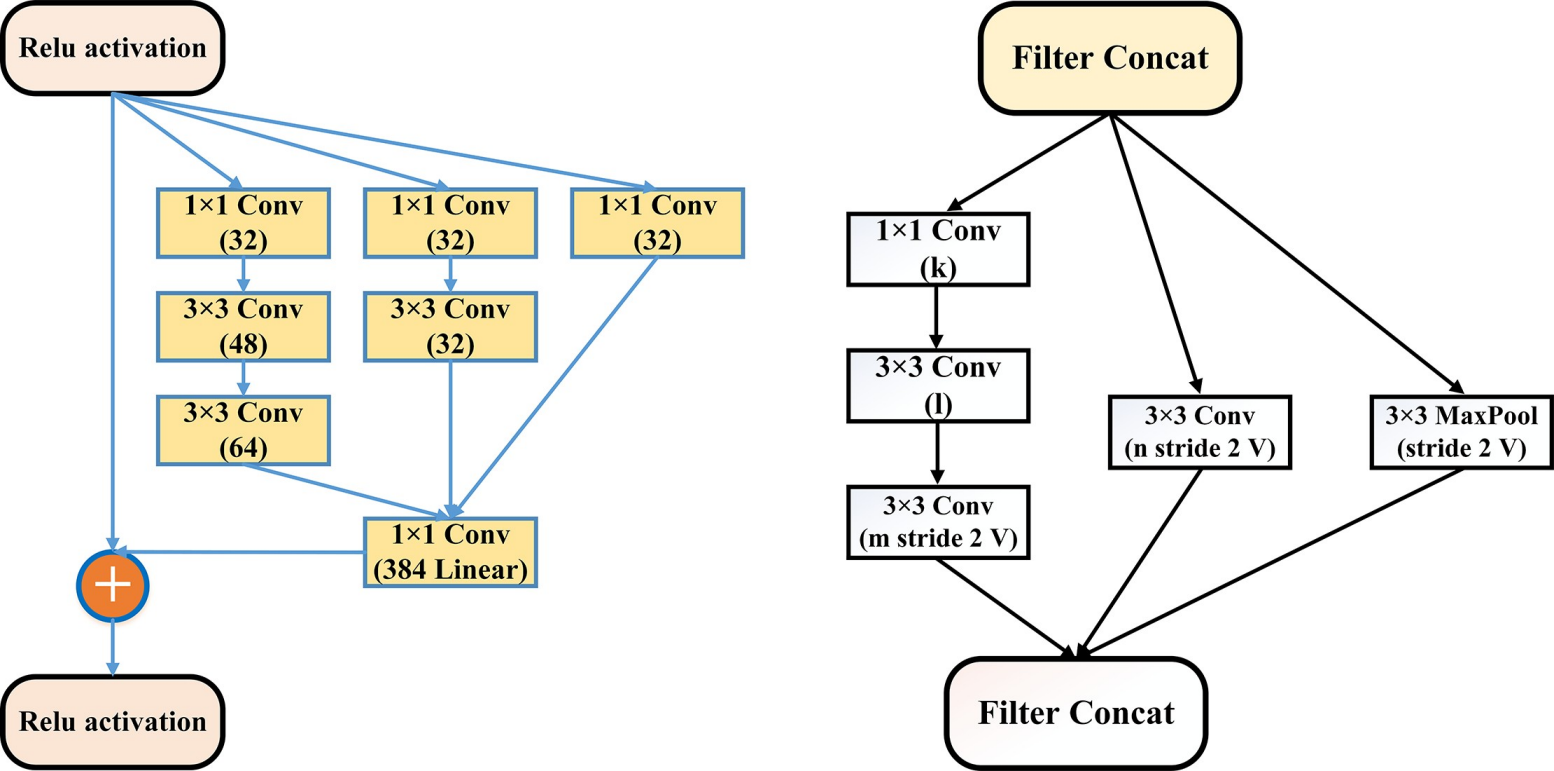
Illustration of CO-IRv2 (a) Inception-A module (b) Reduction-A module.

Fig 5(A) and 5(B) depict the detailed architecture of Inception-A, and Reduction A., respectively in CO-IRv2. In the Inception-A module, there are three paths of 1×1 and 3×3 convolution operations. The illustrations of Inception-Res-Net B, Reduction B, Inception-Res-Net C modules are similar to those reported in [56]. The Inception-ResNet-B and Inception-ResNet-C have two paths of convolution operations with different convolution filter sizes. Each inception block is connected to a filter layer, a form of 1 × 1 convolution operation. This filter layer obtains transformation in dimensions for matching with the input. This compensates for the reduction in dimensions at the inception stage. Inception-A module shown in Fig 5(A) is created for 35×35 grid blocks of the traditional Inception-v4 network. Conversely, Inception-B module has 17×17 grid modules and Inception-C has 8×8 grid blocks. There are also Reduction-A and Reduction-B layers. The Reduction-A block has one path for max-pooling and two paths for convolution operation. The Reduction-B block consists of three convolution paths and one max-pooling path. The Reduction-A module shown in Fig 5(B) reduces 35×35 to 17×17 modules. Moreover, the Reduction-B module reduces 17×17 modules to 8×8 grid.

A number of optimizers namely Adam, Nadam and RMSprop are used for CO-IRv2. Adam is a method for calculating adaptive learning rates. It keeps an exponentially decaying average of past squared gradients as well as an exponentially decaying average of past gradients like momentum. Nadam is a combination of Adam and Nesterov accelerated gradient (NAG). The NAG term is incorporated in Adam by changing the momentum term achieved by first moving in the direction of the previous momentum vector and then moving in the direction of the current gradient. RMSprop is a gradient-based optimization strategy suitable for neural network training. Table 3 presents the parameters of optimizers such as Adam, Nadam and RMSProp with learning rates. CO-IRv2 has 56,444,642 parameters for building the model where the number of non-trainable parameters is 54,340,832 and the number of trainable parameters is 2,103,810.

**Table 3:** Optimizers with various parameters for training and testing of CO-IRv2.

Optimizers	Learning Rate	Other Parameters
Adam	0.002	beta_1 = 0.9,
		beta_2 = 0.999,
		epsilon = 1e-07,
Nadam	0.001	beta_1 = 0.9, beta_2 = 0.999, epsilon = 1e-07,
RMSProp	0.001	rho = 0.9,
		momentum = 0.1,
		epsilon = 1e-07

## 5. Experimental results and discussion

In this section, the experimental results of CO-IRv2 are first described for the case of CT images and then for X-ray images.

### 5.1 Results for CT images

This section discusses the experimentation of CO-IRv2 applied to CT images. Google Collaboratory was used for experimental purposes. This is a cloud service based on Jupyter notebook for distributing knowledge and applying DL. It deals with a fixed GPU with free-of-charge and fully optimized runtime for DL or ML. In the experiments, Tensorflow, Matplotlib, Sklearn and Numpy were also applied as libraries. In the following, the performance metrics are described and then the results are presented.

Firstly, several performance metrics, including confusion matrix, precision, recall, accuracy, F1-score, ROC curve [11–13, 57–59] are defined in the context of CO-IRv2 model. The result of a suspected patient is negative if the lung is not infected from coronavirus and is positive when the coronavirus has infected the lungs. The outcome of this test for all COVID-19 patients may not or may match the actual cases of the patients. As an element of the confusion matrix, the true positive (TP) denotes the positive COVID-19 patients correctly identified. Another term called false positive indicates incorrectly identified COVID-19 patients who may or may not have other lung diseases. At the same time, true negative (TN) means correctly detected COVID-19 negative patients. False-negative represents the incorrectly identified non-infected patients. Classification accuracy is the measure of the correctness of identifying a normal case as normal and an abnormal cases as abnormal. Accuracy, *A*, is given as.


$$A=\frac{TP+TN}{TP+TN+FP+FN}$$ (3)

Recall is the number of correctly classified patients to the number of suspected patients. Recall or sensitivity, *R*, is given by.


$$R=\frac{TP}{TP+FN}$$ (4)

Specificity, *S*, refers to the prediction accuracy of normal cases expressed as

$$S=\frac{TN}{TN+FP}$$ (5)

Precision, *P*, is the ratio of accurately classified positive cases to the overall predicted positive cases given by.


$$P=\frac{TP}{TP+FP}$$ (6)

F1-score, *F*_1_, is the harmonic mean of precision, *P*, and recall, *R*, calculated as follows.


$$F_{1}=\frac{2\times P\times R}{P+R}$$ (7)

**Fig 6:**
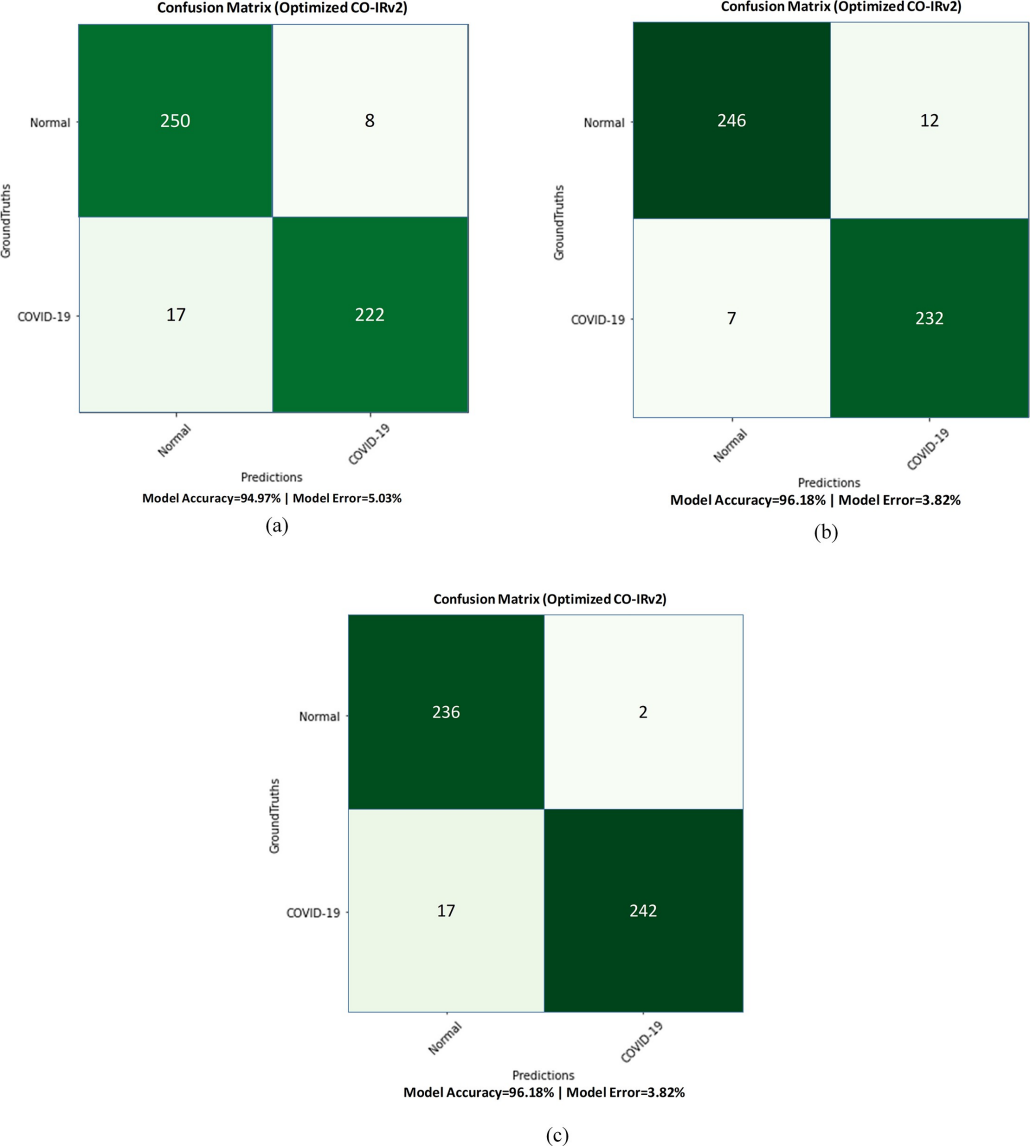
Confusion matrix of CO-IRv2 for (a) Adam, (b) Nadam and (c) RMSProp.

Next, results are provided for the case where holdout method is used to separate training and testing images. Results are for the case of 25 epochs. Fig 6 shows the values of the confusion matrix of CO-IRv2 for Adam, Nadam and RMSProp. It can be seen from Fig 6 that the values achieved for TP, TN, FN, FP are 250, 222, 17, 8, respectively, for Adam optimizer. Moreover, for the case of Nadam optimizer, TP, TN, FN, FP have values of 246, 232, 7, 12, respectively. Furthermore, the values achieved for TP, TN, FN, FP are 236, 242, 17, 2, respectively, for RMSProp optimizer in CO-IRv2. The obtained accuracies of Adam, Nadam and RMSProp optimizers are 94.97%, 96.18% and 96.18%, respectively. This is shown in Table 4. For Nadam and RMSProp optimizers, our proposed model obtains the highest accuracy where the accuracy of normal and COVID-19 is 96.18% individually. Table 4 shows that Nadam provides a recall value of 97% normal and 95% COVID-19, whereas RMSProp has a recall value of 93% normal and 99% COVID-19.

**Table 4:** Classification of COVID-19 and normal patients for different optimizers using CO-IRv2.

Optimizer	Class	Sensitivity/Recall	Precision	F1-Score	Accuracy
Adam	Normal	94%	97%	95%	94.97%
	COVID-19	97%	93%	95%	94.97%
Nadam	Normal	97%	95%	96%	96.18%
	COVID-19	95%	97%	96%	96.18%
RMSProp	Normal	93%	99%	96%	96.18%
	COVID-19	99%	93%	96%	96.18%

**Fig 7:**
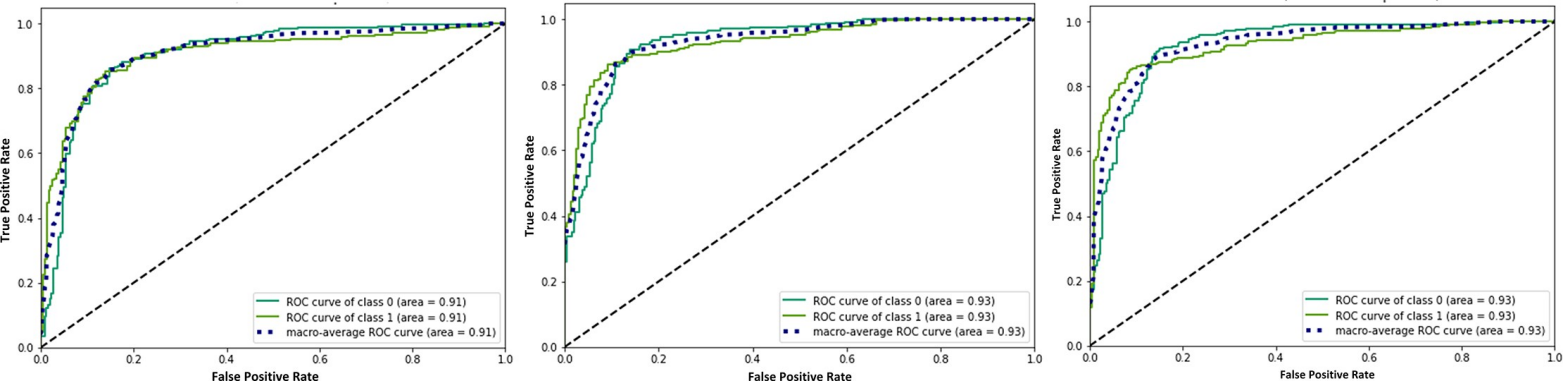
ROC plot of InceptionNetV3 for the optimizer of (a) Adam, (b) Nadam and (c) RMSProp.

**Fig 8:**
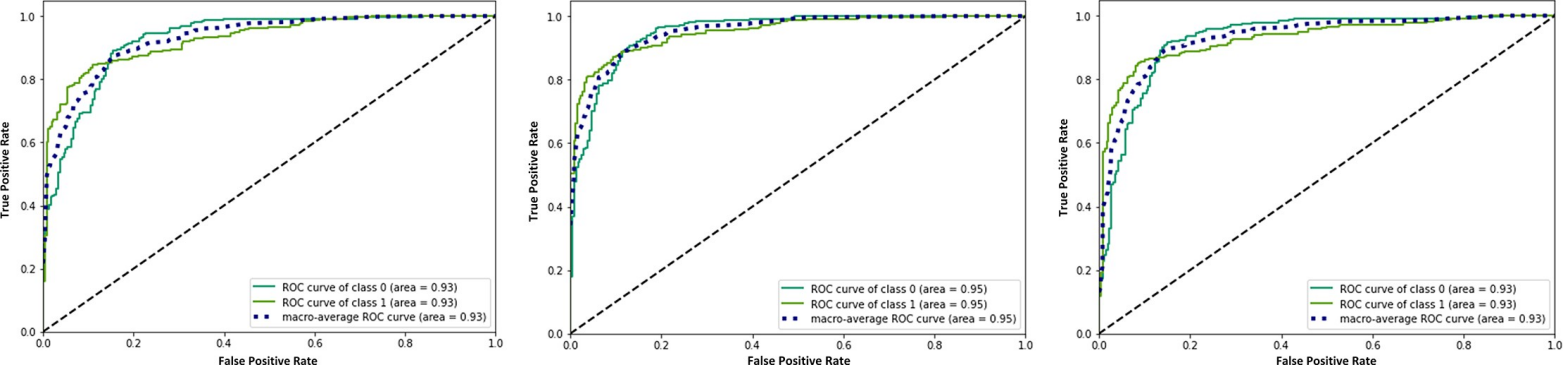
ROC plot of CO-IRv2 for the optimizer of (a) Adam, (b) Nadam and (c) RMSProp.

**Fig 9:**
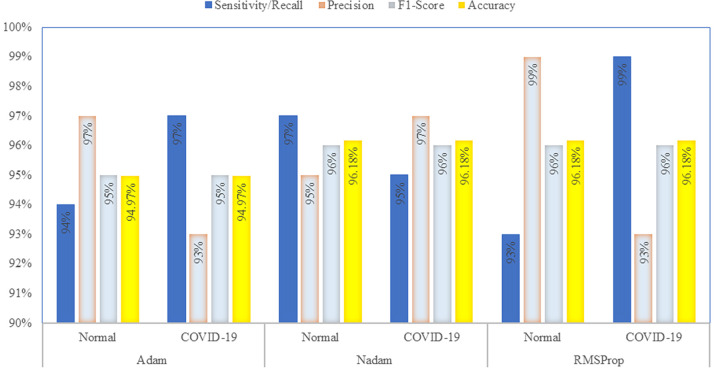
Comparison of different optimizers for CO-IRv2 model for each classes.

The efficiency of CO-IRv2 is also apparent from the AUC-ROC curves for CO-IRv2 and those of existing InceptionNetV3 model. The ROC curves in Fig 7 depict the performance of InceptionNetV3 for different optimizers, while Fig 8 presents that of CO-IRv2. Fig 7 shows that in the case of InceptionNetV3, the AUC values for Adam, Nadam and RMSProp are 91%, 93% and 93%, respectively. From Fig 8 it can be seen that for the case of CO-IRv2, the AUC values of Adam, Nadam and RMSProp CO-IRv2 are 93%, 95% and 93%, respectively. Hence, for each optimizer, CO-IRv2 has higher AUC values than InceptionNetV3. Furthermore, it can be seen that the Nadam optimizer is more efficient than other optimizers when CO-IRv2 is applied to diagnose the infection of COVID-19. Fig 9 compares different optimizers for CO-IRv2 model for each class. Fig 9 presents that for CO-IRv2, the classification accuracy is higher for RMSProp and Nadam compared to Adam optimizer.

**Fig 10:**
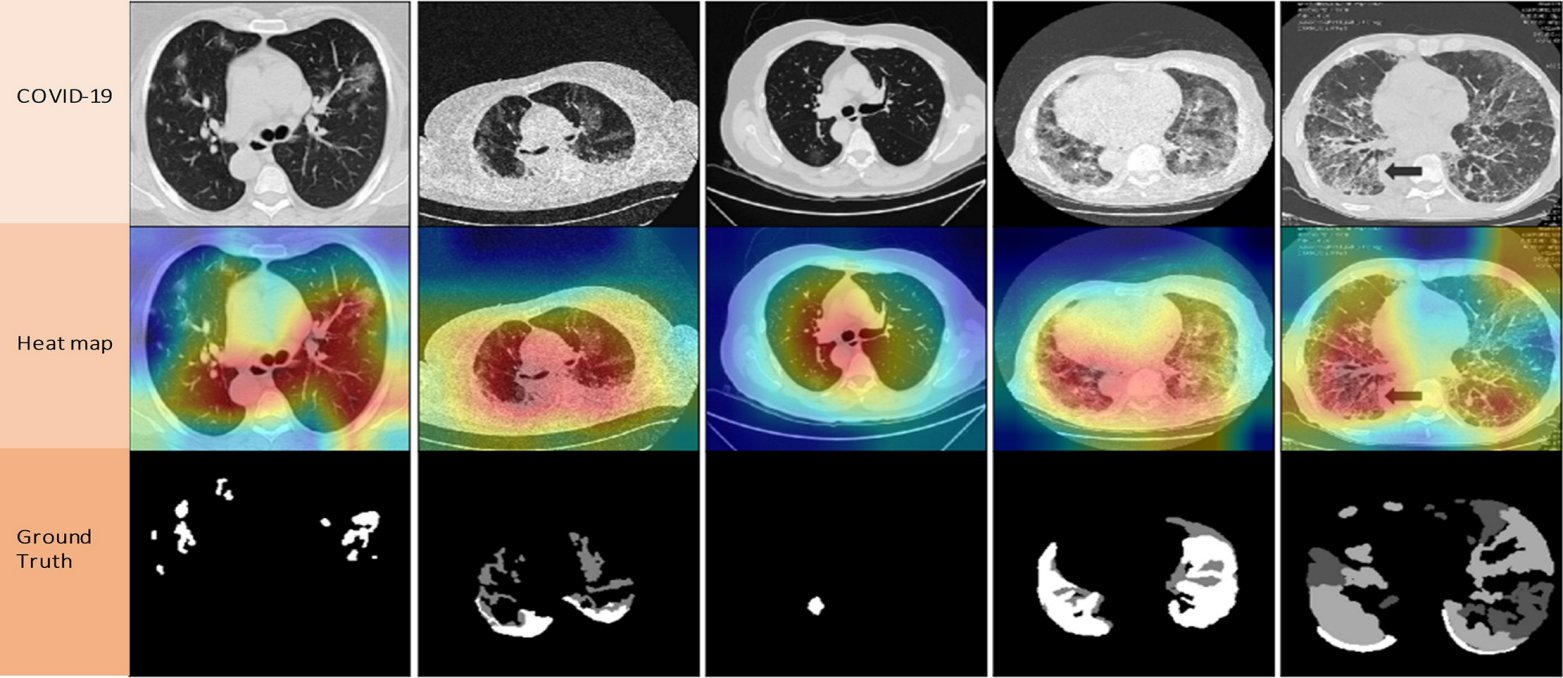
Ground truth and heatmap of COVID-19 CT images when CO-IRv2 is used.

Next, heatmap is used for evaluating the performance of CO-IRv2. It visualizes the operation of the proposed algorithm. Fig 10 visualizes ground truth and heatmap of COVID-19 CT images when CO-IRv2 is used. The ground truth achieved from the heatmap indicates the actual results of the detection of COVID-19 infection.

In the experiments, the performance of CO-IRv2 was also evaluated using the cross-validation method to separate the training and testing images in the dataset. For this, five-fold cross-validation was chosen. Since Nadam optimizer exhibited the best results for the holdout method for CT images, we used Nadam for cross-validation. The total images were separated into five groups marked as groups 1–5. For the first fold, the images from group 1 were used for testing, while the images from groups 2–5 were used for training. With this consideration, the classification accuracy and other metrics were calculated for the first fold. Next, group 4 was used for testing, while the images from groups 1–3 and group 5 were used for training. This arrangement enabled the calculations for the second fold. Similarly, the third fold, fourth fold, and fifth fold metrics were calculated considering group 3, group 4, and group 5 for testing, respectively, with the remaining ones used as training images. The metrics obtained for folds 1–5 were averaged to obtain the final results. The obtained values for precision, recall, specificity, F1-score and accuracy were 97.85%, 91.20%, 97.96%, 94.41% and 94.55%, respectively.

### 5.2 Comparison of CO-IRv2 with existing methods for CT images

This section provided comparative results of the proposed CO-IRv2 with the existing methods for the case of CT images. Table 5 shows comparative results of CO-IRv2 with the existing InceptionNetV3 model for Adam, Nadam and RMSProp optimizers. Table 5 also indicates that compared to InceptionNetV3, our proposed CO-IRv2 model achieves higher values of sensitivity, precision, recall, F1-score and accuracy. The execution time of CO-IRv2 is comparable to that of InceptionNetV3 for each of the optimizers. Table 6 compares the execution time for CO-IRv2 with several existing models. CO-IRv2, VGG-19, CTnet-10, and Inception V3 models were trained and evaluated using a Tesla K80 Graphical Processing Unit (GPU) supplied by Google Colab, while DenseNet-169 model was run using an eighth-generation Intel i5 CPU. On the other hand, MobileNet, VGG16, DenseNet121, DenseNet169, NasNet Large, Xception and InceptionResNetV2 were run using the Google Collaboratory with Tesla K80 GPU Card in conjunction with an Intel i7-core @3.6 GHz processor, 16 GB RAM and 64-bit Windows 10 operating system. From Table 6, it can be seen that CTnet-10 has a low execution time of 130.90 seconds, while running on Tesla K80 GPU. On the other hand, MobileNet has a low execution time of 374 seconds while running on a Tesla K80 GPU Intel i7-core @3.6 GHz processor and 16 GB RAM. The proposed CO-IRv2 has an execution time of 707 seconds.

**Table 5:** Comparison of CO-IRv2 with existing InceptionNetV3 model.

Model	Optimizer	Precision	Recall	F1-Score	Specificity	Accuracy	Execution Time (sec)
InceptionNetV3	Adam	90.76%	87.10%	88.89%	91.20%	89.16%	730
	Nadam	93.70%	90.28%	91.96%	94.00%	92.15%	598
	RMSProp	94.96%	89.33%	92.06%	95.08%	92.15%	712
CO-IRv2	Adam	96.90%	93.63%	95.24%	96.52%	94.97%	717
	Nadam	95.35%	97.23%	96.28%	95.08%	96.18%	707
	RMSProp	99.16%	93.28%	96.13%	99.18%	96.18%	749

**Table 6:** Computational time of various models for CT scans of COVID-19 patients.

Models	Machine Used	Execution time (sec)	Reporting Ref.
DenseNet-169	eighth generation Intel i5 CPU	448.73	[32]
VGG-19	Tesla K80 GPU	514	[32]
CTnet-10		130.90	
MobileNet	Tesla K80 GPU Intel i7-core @3.6 GHz processor and 16 GB RAM	374	[60]
VGG16		618	[60]
DenseNet121		989	[60]
NasNet Large		2170	[60]
Xception		795	[60]
InceptionResNetV2		1369	[60]
InceptionNetV3 (Nadam)	Tesla K80 GPU	598	This paper
CO-IRv2 (Nadam)	Tesla K80 GPU	707	This paper

Table 7 briefly compares CO-IRv2 model with the state-of-art methods in terms of recall, precision, recall, F1-score and accuracy. Our proposed CO-IRv2 model achieves the highest accuracy and recall when compared with the existing models in Table 7. The accuracy obtained by CO-IRv2 outperforms all referred models taken into consideration. The accuracies described by Ref. [30, 42, 43, 46] are around 86% for CT images. The ElasticNet [47] and UNet [41] models have accuracy values of 90% and 91.66%, respectively. However, the accuracy of CO-IRv2 is 96.18%. Hence, CO-IRv2 has the potential to be considered as an efficient system for binary classification of lung CT images.

**Table 7:** Comparison of the proposed method with a number of existing literature.

References	Methods	AUC	Precision	Specificity	Recall	F1-Score	Accuracy
[33]	U-Net CNN	95.90%	-	91.10%	90.70%	-	-
[34]	2D CNN	-	-	88%	87%	-	89.50%
[29]	ResNet50	81.90%	-	61.50%	81.10%	-	76%
[30]	ResNet18	-	80.80%	-	81.50%	81.10%	86.70%
[41]	BCDU-Net (UNet)	-	-	94%	87.50%	-	91.66%
[42]	CRNet	94%	-	-	-	85%	86%
[43]	Multi-layer perceptron with Encoder Decoder	93%	-	79%	94%	-	86%
[46]	Multi-task learning and single-task learning	86%±2%	-	88%±1.50%	77%±3%	-	86%±2%
[47]	Elastic Net	94%	-	91%	79%	-	90%
**Proposed CO-IRv2**	Adam	93%	96.90%	96.52%	93.63%	95.24%	94.97%
	Nadam	95%	95.35%	95.08%	97.23%	96.28%	96.18%
	RMSProp	93%	99.16%	99.18%	93.28%	96.13%	96.18%

### 5.3 Results for X-ray images

This section provides brief results of CO-IRv2 applied to a dataset of X-ray images [54]. The dataset contains 1662 images of which 1583 images are of normal people and 79 are of COVID-19 patients. Using the holdout concept, 80% of the total images are used for training and the remaining 20% for testing. This means 1329 images are for the training, of which 1261 and 68 are for normal and COVID-19 patients, respectively. On the other hand, 333 images are used for testing where 11 are of COVID-19 patients, and the remaining 322 are of normal people. Table 8 shows the performance results for CO-IRv2 with different optimizers for the case of X-ray image dataset. The performance is evaluated using a Tesla K80 GPU supplied by Google Colab. It can be seen from Table 8 that the best results are obtained by Adam optimizer in terms of accuracy, precision, recall, F1-score and specificity. CO-IRv2 with Adam optimizer achieves classification accuracy of 99.40% and a recall value of 99.38% for an execution time of 430 seconds. The classification accuracy value of Adam is better than Nadam and RMSProp. However, Nadam and RMSProp optimizers have slightly lower execution times than Adam. The results obtained for CO-IRv2 are comparable to the results reported in the literature of X-ray images. For example, the studies in [16, 48–50] consider different X-ray datasets and report accuracy values of 96.10%, 98.75%, 98.66% and 98.08%, respectively. On the other hand, CO-IRv2 with Adam optimizer can obtain an accuracy of 99.40% for the case of X-ray images.

**Table 8:** Comparison of CO-IRv2 for different optimizer using X-ray images.

Model	Optimizer	Precision	Recall	F1-Score	Specificity	Accuracy	Execution Time (sec)
CO-IRv2	Adam	100%	99.38%	99.69%	100%	99.40%	430
	Nadam	99.07%	99.38%	99.22%	75.00%	98.50%	417
	RMSProp	100%	99.08%	99.54%	100%	99.10%	428

### 5.4 The implication of the results

It is shown in this paper that CO-IRv2 can be successfully applied to the images of CT scans and X-ray for COVID-19 screening. Processes involving Image testing and clinical testing are different. A clinical test can be unpleasant compared to imaging trials, which do not require a lengthy procedure. Clinical testing has slightly higher accuracy when doctors analyze the reports themselves, and in the field of image testing, a computer does this until detailed instructions are given. Image analysis is less expensive than clinical analysis. However, CT scan radiation can be a concern particularly for pregnant women and those with metal implants. CO-IRv2 approach classifies CT scans as COVID-19 negative or positive. We may extend our CO-IRv2 model further to detect positive COVID-19 CT scan images, depending on the extent of COVID-19 dissemination in the pulmonary area. Our method is well organized and can be used by doctors to screen large numbers of individuals. It is likely to be comparable with the accuracy and speed of the current RT-PCR method in COVID-19 diagnosis. The CO-IRv2 approach can categorize CT scan images of COVID-19 patients, allowing doctors to obtain data efficiently and timely.

## 6 Conclusions and future work

This paper introduces and describes a new scheme termed as CO-IRv2. This new CO-IRv2 scheme has two major blocks. One is the combination of the idea of InceptionNet and ResNet, while the other is a combination of a global average pooling layer, batch normalization, dense layers, and dropout layers. This CO-IRv2 is suitable for diagnosing and evaluating each class (normal vs. COVID-19) by applying different optimizers such as Adam, Nadam and RMSProp. The proposed model is applied to a new dataset of CT images which is formed here as the combination of two databases. A number of data preprocessing techniques, including data segmentation, augmentation, rescaling and data normalization are applied to the new dataset. For CO-IRv2, hyperparameters fine-tuning are applied for optimization. For the case of CT images, CO-IRv2 achieves the highest 96.18% accuracy, 97.23% recall, 95% AUC and 96.28% F1-score for Nadam optimizer. The proposed CO-IRv2 is also applied to an X-ray dataset of 1662 images. It is found that CO-IRv2 with Adam optimizer achieves classification accuracy of 99.40% and a recall value of 99.38%.

The proposed CO-IRv2 model can be executed in the medical field to classify chest X-ray and CT images to find out the COVID-19 patients. This can be utilized as a pre-assessment method for shortening the workload of physicians and doctors, diagnosing and treating diseases at initial stages. This method can be used to determine the condition of the patients before applying treatment quickly. Although CO-IRv2 can predict COVID-19 patients, the effectiveness of the method depends on the datasets. If a dataset contains incomplete labels or the images have significant distortions and noise, the disease prediction may be inaccurate. Like other DL methods, CO-IRv2 requires large-sized reliable data for training so that the prediction can be reliable. There is a limited number of large and balanced datasets. Moreover, there is no benchmark dataset for COVID-19, so it is challenging to compare CO-IRv2 with others in a precise way. Nevertheless, in the future, the effectiveness of CO-IRv2 should be evaluated for large datasets of COVID-19.
